# Effects of Maillard Reaction Durations on the Physicochemical and Emulsifying Properties of Chickpea Protein Isolate

**DOI:** 10.3390/foods14010117

**Published:** 2025-01-03

**Authors:** Shanshan Zhang, Yibo Liu, Wenhui Wu

**Affiliations:** 1 Department of Marine Biopharmacology, College of Food Science and Technology, Shanghai Ocean University, Shanghai 201306, China; zss13139939001@163.com; 2 School of Food Science and Technology, Shihezi University, Shihezi 832003, China; lyb14235@163.com; 3 Marine Biomedical Science and Technology Innovation Platform of Lin-Gang Special Area, Shanghai 201306, China

**Keywords:** chickpea protein isolate, physicochemical characteristics, emulsifying properties, Maillard reaction

## Abstract

This study investigated the physicochemical and emulsifying properties of chickpea protein isolate (CPI)-citrus pectin (CP) conjugates formed via the Maillard reaction across varying reaction durations. CPI and CP were conjugated under controlled dry-heating conditions, and the resulting conjugates were characterized by measuring their particle size, zeta potential, solubility, thermal stability, surface hydrophobicity, and emulsifying properties. The results showed that as reaction duration increased, the particle size and zeta potential of the CPI-CP conjugates increased significantly, reaching a maximum particle size of 1311.33 nm and a zeta potential of −35.67 mV at 12 h. Moreover, the Maillard reaction improved the solubility, thermal stability, and hydrophobicity of the CPI. Glycosylation increased the emulsifying activity index (EAI) and emulsifying stability index (ESI) of the CPI to 145.33 m^2^/g and 174.51 min, respectively. Optimal emulsions were achieved at a protein concentration of 1.5 wt% and a 10% volume fraction of the oil phase. The Maillard reaction promoted the interfacial protein content and the thickness of the interfacial layer while decreasing the droplet size and zeta potential of the emulsion. Additionally, the emulsion prepared with CPI-CP-12 h showed outstanding long-term stability. These results demonstrate that a moderate Maillard reaction with CP effectively enhances the physicochemical and emulsifying characteristics of CPI.

## 1. Introduction

In response to the increasing global emphasis on sustainable food resources, plant proteins have gained attention as vital alternatives to animal proteins, becoming central to research and development in the food industry [1]. As the population of the world grows and pressures on animal-based food production escalate, the development and enhancement of plant proteins are becoming increasingly urgent [2,3]. Among the various plant protein sources, chickpea protein isolate (CPI) stands out for its excellent nutritional profile and broad potential applications [4]. CPI contains abundant essential amino acids, especially lysine and arginine, and it is naturally free of cholesterol and lactose, making it an ideal substitute for dairy and meat proteins [5]. Additionally, as a legume, chickpeas have low allergenic potential, further enhancing the suitability of CPI in dairy-free and gluten-free markets [6]. This unique profile establishes the protein isolate of chickpeas as a valuable ingredient for health-oriented and functional foods, including beverages, meal replacements, baked goods, and snacks [7].

Despite the excellent nutritional profile of CPI, several challenges limit the broader application of CPI in food products. CPI may have a beany or bitter flavor, which could impact the overall taste of the food and affect consumer acceptance [8]. The molecules of CPI have a compact structure maintained by strong hydrogen bonds and hydrophobic interactions, which restrict their unfolding and solubility in aqueous environments [9]. During high-temperature processing, CPI is prone to irreversible denaturation, resulting in decreased functionality, particularly with respect to heat stability and texture [4]. This denaturation restricts the applicability of CPI in high-temperature processes, such as baking and the production of ready-to-eat meals. Furthermore, the high proportion of hydrophobic amino acids and limited exposure of hydrophilic groups in the amino acid profile of CPI reduces its stability at the oil/water interface, hindering the composition of stable emulsions [10]. Therefore, modification strategies to improve the functional properties of plant proteins have gained significant research attention.

The Maillard reaction is a non-enzymatic browning process between amino groups in proteins and carbonyl groups in reducing sugars, leading to covalent conjugates [11]. The reaction proceeds without the need for additional chemical reagents, offering the benefits of environmental safety and mild reaction conditions [12]. Additionally, Maillard reaction products possess unique aromas and flavors, which can help mitigate the undesirable odors of proteins to some extent. Therefore, the Maillard reaction offers an effective strategy for enhancing the physicochemical and functional properties of proteins, thereby expanding their potential applications in the food industry [13]. Research has demonstrated that the Maillard reaction effectively improves plant protein functionality. For example, it significantly increased the water solubility of soy protein isolate by adding hydrophilic groups from sugar molecules to the surface of the protein [14]. After undergoing the Maillard reaction, the oyster protein hydrolysates showed a reduction in unsaturated aldehydes and ketones responsible for fishy and bitter odors, while compounds contributing to meaty aromas increased [15]. Xylo-oligosaccharides can interact with pea protein amino acid residues to form a protective layer, preventing structural unfolding and degradation under high temperatures [16]. Additionally, glycosylation increased the emulsification of coconut protein by decreasing interfacial tension and enhancing its adsorption at the interface [17]. Protein-polysaccharide conjugates also stabilize emulsions by increasing steric hindrance, which reduces droplet aggregation and rupture [18].

Polysaccharides possess large and highly branched molecular structures, enabling them to form dense interfacial layers with protein molecules at the oil-water interface [19]. This enhances their adsorption capacity and emulsification stability compared to monosaccharides and oligosaccharides [20]. Furthermore, the molecular structure of polysaccharides not only provides physical protection for proteins but also prevents heat-induced protein aggregation through interactions with the amino acid side chains [21]. Citrus pectin (CP), a natural polysaccharide primarily composed of polysaccharide chains linked by α-1,4-glycosidic bonds between galacturonic acid units, is rich in carbonyl and hydroxyl functional groups [22]. These groups interact with protein amino acid residues, thereby facilitating the Maillard reaction. In a previous study, we investigated the effects of different Maillard reaction durations on the structure and oil-water interfacial behavior of CPI [23]. However, research on the conjugation of CPI with CP remains limited, particularly regarding functional enhancement through the Maillard reaction.

This study employed CP as a reducing polysaccharide to combine with CPI through the Maillard reaction, intending to improve the functional properties of CPI. This research evaluated the particle size, zeta potential, solubility, thermal stability, and emulsification of both CPI and CPI-CP conjugates. Additionally, it measured the droplet size, zeta potential, apparent viscosity, and long-term stability of the emulsions. This study proposes an innovative strategy for modifying CPI to broaden its applications within the food industry.

## 2. Materials and Methods

### 2.1. Materials

Chickpea protein isolate (CPI, protein content > 85%, derived from Kabuli-type chickpea) was sourced from Mulei Yingge Bio-technology Co., Ltd. (Changji, China). Citrus pectin (CP, galacturonic acid ≥ 74.0%, degree of esterification: 73%) was obtained from Sigma-Aldrich (St. Louis, MO, USA). Sunflower seed oil was supplied by Yihai Kerry Co., Ltd. (Hangzhou, China).

### 2.2. Preparation of CPI-CP Conjugates

A 1:2 mass ratio mixture of CPI and CP was dissolved in a 10 mM phosphate buffer (pH 7.0). The solution was freeze-dried for 48 h and then ground into a fine powder. The resulting powder was incubated in a temperature- and humidity-controlled chamber at 70 °C and 79% relative humidity for a duration of 0 to 36 h [23].

### 2.3. Measurement of Protein Particle Size and Zeta Potential

Samples were prepared by dissolving in a 0.01 M phosphate buffer at pH 7.0, followed by equilibration at 25 °C for 2 min before analysis. Particle size was measured using a Zetasizer (Advance Pro, Malvern Instrument Ltd., Malvern, UK) with a DTS0012 sample cell, holding 1 mL of solution and employing a 173° backscattering detection angle. Zeta potential was determined in a DTS1070 U-shaped cell, with three measurements taken for each sample to ensure accuracy.

### 2.4. Measurement of Solubility

A protein solution (1.0 mg/mL) containing CPI and CPI-CP conjugates was adjusted to pH 7.0 with 0.1 M NaOH and stirred for 1 h. The solution was centrifuged at 5600× *g* for 15 min at 4 °C. The protein concentration in the supernatant was measured using the Bradford method with a standard curve based on BSA [24]. Protein solubility was calculated using the following equation:

(1) $$\mathrm{Solubility}\;\left(\%\right)=\frac{C_{S}}{C_{T}}\times 100$$

where *C_S_* represents the protein concentration in the supernatant (mg/mL), and *C_T_* represents the total protein concentration (mg/mL).

### 2.5. Determination of Thermal Stability

The thermal stability of samples was evaluated following established protocols [25]. Differential scanning calorimetry (DSC) was conducted with a 209 F1 (NETZSCH Instrument Ltd., Selb, Germany) from 20–200 °C, and thermogravimetric analysis (TG) was performed using an STA 449 F1 (NETZSCH Instrument Ltd., Selb, Germany) over a temperature range of 50–550 °C. Each 5 mg sample was placed in a crucible, with an empty crucible serving as the reference.

### 2.6. Determination of Emulsifying Activity Index (EAI) and Emulsifying Stability Index (ESI)

The EAI and ESI of the protein samples were assessed using the method outlined by Ma et al. [26]. The protein solution was combined with sunflower seed oil at a 3:1 volume ratio and homogenized at 10,000 rpm for 3 min. At 0 and 10 min, 50 μL of the lower emulsion layer was diluted in 5 mL of 0.1% SDS solution, and the absorbance was immediately measured at 500 nm. EAI and ESI were calculated using the following formulas:

(2) $$\mathrm{EAI}\;(m^{2}/g)=\frac{2\times 2.303\times A_{0}\times N}{10,000\times \theta \times L\times C}$$

(3) $$\mathrm{ESI}\;\left(\min\right)=\frac{A_{0}\times 10}{A_{0}-A_{10}}$$

where *N* represents the dilution factor, *θ* denotes the volume ratio of the oil phase, *L* is the path length of the cuvette in cm, and *C* indicates the protein concentration in mg/mL. *A*_0_ and *A*_10_ are the absorbances measured at 0 min and 10 min, respectively, with 10 representing the time interval in minutes between the two measurements.

### 2.7. Preparation of Emulsion

The samples were dissolved in 0.01 M PBS (pH 7.0) containing 0.02 wt% sodium azide and stirred overnight. Sunflower seed oil was added at various volume fractions (5%, 10%, 15%, 20%, 25%, and 30%), and a coarse emulsion was prepared using a high-speed shear mixer at 20,000 rpm for 2 min. The emulsion was then refined using a high-pressure homogenizer at a pressure of 500 bar for two passes [23].

### 2.8. Measurement of Emulsion Droplet Size

The droplet size and droplet size distribution of the emulsions were assessed using a laser particle size analyzer (Mastersizer 3000+ Lab., Malvern Instrument Ltd., Malvern, UK). The refractive indices of sunflower seed oil and water were set at 1.47 and 1.33, respectively [27].

### 2.9. Measurement of Emulsion Zeta Potential

The zeta potential of the emulsions was measured via microelectrophoresis using a Zetasizer (Advance Pro, Malvern Instrument Ltd., Malvern, UK). Results were obtained from three independent measurements conducted at 25 ± 0.02 °C.

### 2.10. Percentage of Interfacial Adsorbed Protein and Thickness of the Adsorbed Layer

Fresh emulsions were centrifuged at 11,000× *g* for 30 min. After centrifugation, the serum layer at the bottom of the tube was collected with a syringe. Protein concentrations in both the serum and the initial emulsion were determined using the Kjeldahl method. The percentage of adsorbed protein (AP) was evaluated using the following formula [28]:

(4) $$\mathrm{AP}\;\left(\%\right)=\frac{C_{E}-C_{S}}{C_{E}}\times 100\%$$

where *C_S_* is the protein concentration of the serum, and *C_E_* is the initial protein concentration of the emulsion.

To prepare the original solution with the same emulsifier concentration as in Section 2.7, the method by Lin et al. [29] was used. A nano-particle size analyzer was used to mix 2440 μL of emulsion with 60 μL of 2.5% *w*/*v* monodisperse polystyrene latex beads. After allowing the mixture to reach adsorption equilibrium for 8 h, the particle size difference before and after adsorption onto the polystyrene latex beads was measured to calculate the interfacial layer thickness:

(5) $$d_{T}\;(\mathrm{nm})=\frac{R_{a}-R_{i}}{2}$$

where *d_T_* is the interfacial layer thickness, and *R_a_* and *R_i_* represent the particle sizes of the polystyrene latex beads when protein is adsorbed and not adsorbed, respectively.

### 2.11. Viscosity of the Emulsion

The apparent viscosity of the fresh emulsion was measured using a rotational rheometer equipped with a double-gap measuring system. Here, at shear rates ranging from 0.1 to 100 s^−1^, the emulsion was allowed to equilibrate at 25 ± 0.02 °C for 10 min before testing [27].

### 2.12. Long-Term Stability of the Emulsion

The long-term storage stability of the emulsion was assessed using a LUMiSizer (LUM GmbH, Berlin, Germany). The wavelength for light detection was set to 870 nm, with a rotation speed of 2828 rpm and a temperature of 25 °C. Scanning was performed every 15 s for a total of 1000 scans. Transmission curves for each sample tube were recorded as separation progressed.

### 2.13. Statistical Analysis

All experiments were conducted in triplicate, and the data are presented as the mean ± standard deviation. Data were analyzed by one-way analysis of variance (ANOVA) using SPSS 27 (SPSS Inc., Chicago, IL, USA) followed by Duncan’s test to determine statistical differences at the *p* < 0.05 significance level.

## 3. Results and Discussion

### 3.1. Particle Size and Zeta Potential Analysis

**Figure 1:**
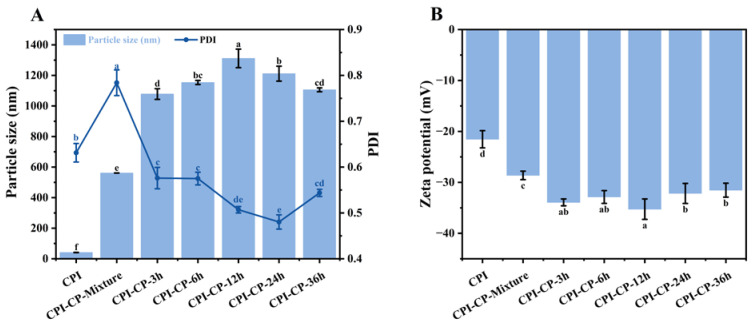
Particle size (**A**) and zeta potential (**B**) of CPI and CPI-CP conjugates. Different letters indicate significant differences (*p* < 0.05).

The particle sizes of the CPI before and after the glycosylation reaction indicate the aggregation state of the proteins. With increasing the reaction time, the CPI-CP conjugates showed a significant enlargement in particle size, reaching a maximum of 1311.33 nm at 12 h (*p* < 0.05) (Figure 1A). This size increase likely reflects the larger solute molecules formed by the interaction of the CPI with the pectin, as well as the covalent bonding between the CPI and the CP, which further enhanced protein particle size. This effect can likely be explained by glycosylation-induced improvements in protein–protein interactions, which promote aggregation. A study by Liu et al. [27] demonstrated that increased pectin addition led to larger chickpea protein particles through the Maillard reaction, indicating that similar glycosylation modifications can elevate the particle size of CPI. However, the particle size of the CPI-CP-36 h sample was reduced to 1105.67 nm, possibly due to the formation of advanced Maillard reaction products, such as aldehydes or ketones, which consumed the protein–polysaccharide conjugates at this stage. As the Maillard reaction progressed, increased covalent bonding with pectin resulted in a more uniform particle size and a marked reduction in PDI.

The zeta potential reflects the type and magnitude of charges in a system and provides insight into solution stability [30]. The zeta potential of the CPI was initially −21.5 mV (Figure 1B). The inclusion of CP led to an increase in the negative charge of the CPI-CP-Mixture sample. After glycosylation, the zeta potential further increased to −35.27 mV. This increase in negative charge may be explained by several factors: (1) Citrus pectin contributes additional negative charges after covalent bonding with the protein [31]. (2) The Maillard reaction reduces the quantity of positively charged lysine residues within the protein [32]. (3) Protein molecules unfold during the Maillard reaction, exposing more negatively charged regions [33]. It is well established that a high negative potential is crucial for emulsion stability. The enhanced negative charge on CPI-CP conjugates may facilitate electrostatic repulsion between proteins at the oil–water interface, facilitating the generation of a thicker interfacial film and thereby improving emulsion stability [34].

### 3.2. Solubility Analysis

**Figure 2:**
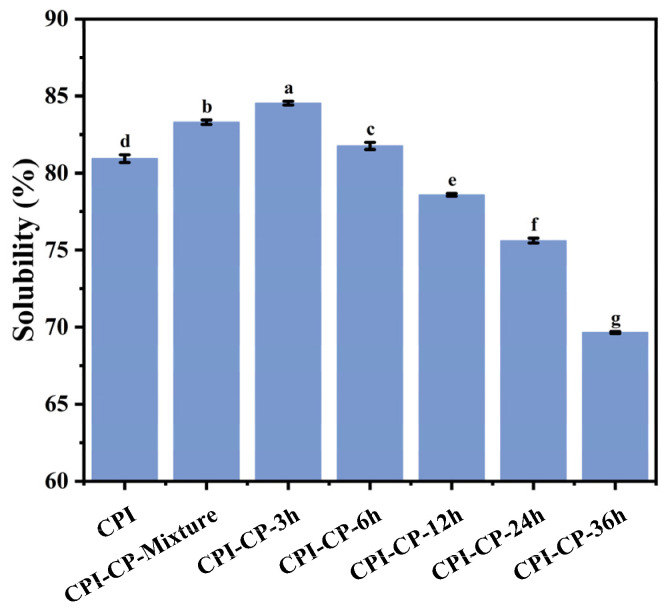
Solubility of CPI and CPI-CP conjugates. Different letters indicate significant differences (*p* < 0.05).

Solubility is a fundamental functional property of proteins, influencing their potential applications. When the reaction duration was less than 3 h, the solubility of the CPI increased to 84.54% (*p* < 0.05) (Figure 2) due to the effect of glycosylation. The enhanced solubility was likely due to the –OH groups in the CP covering the surface of the CPI, enhancing its interaction with water molecules and reducing the surface hydrophobicity [24]. Furthermore, the steric hindrance provided by the CP reduced the interactions between the proteins, further increasing solubility [27]. However, when the reaction time exceeded 3 h, the solubility of CPI exhibited a downward trend. This decline was likely due to the extended heating at 70 °C, which promoted crosslinking and thermal denaturation, exposing internal hydrophobic groups and thereby reducing solubility [20]. Guo et al. [35] similarly found that prolonged heating during Maillard reactions can lower the solubility of whey protein isolate.

### 3.3. Thermal Stability Analysis

**Figure 3:**
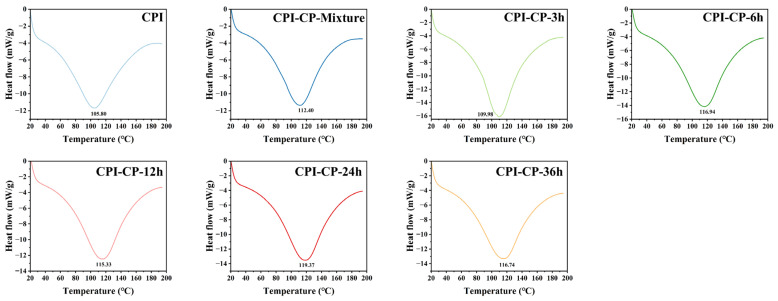
DSC of CPI and CPI–CP conjugates for thermal stability analysis.

The thermal stability, denaturation behavior, and structural changes in the proteins during heating were studied by measuring the heat flow across temperature changes [36]. The differential scanning calorimetry (DSC) results of the CPI and CPI-CP conjugates are shown in Figure 3. The thermal denaturation temperature of the native CPI was 105.80 °C, indicating a thermal transition in the protein. After mixing with CP, the denaturation temperature of the CPI-CP-Mixture sample increased to 112.40 °C, suggesting that the inclusion of the citrus pectin improved the conformational and thermal stability of the CPI. This effect may be attributed to steric hindrance introduced by the CP, which likely restricted protein unfolding. After 3 h of the Maillard reaction, the CPI-CP conjugates exhibited even higher denaturation temperatures, with the CPI-CP-24 h sample reaching 119.37 °C. These results are likely due to strengthened interactions between the pectin and protein molecules. A previous study studied pea protein isolate, where the thermal stability was improved following the Maillard reaction with xylo-oligosaccharide [16].

**Figure 4:**
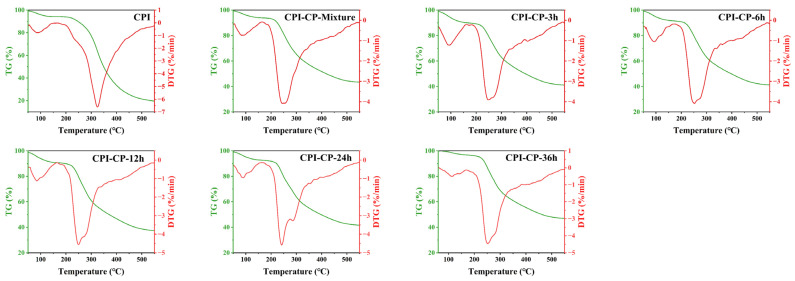
TG and DTG of CPI and CPI-CP conjugates for thermal stability analysis.

Thermogravimetric (TG) and derivative thermogravimetry (DTG) analyses were employed to analyze the thermal stability and decomposition behavior of the proteins. The TG and DTG curves illustrate the stages of protein degradation during heating, providing insights into thermal degradation behaviors such as moisture loss and structural breakdown [25]. The TG curve (green) reflects the mass loss of the sample during the heating, while the DTG curve (red) shows the rate of mass loss at different temperatures (Figure 4). Both the CPI and CPI-CP conjugates exhibited three primary degradation stages: The initial stage occurred between 50–150 °C, associated with moisture evaporation. The second stage, occurring between 200–400 °C, showed a sharp decline in mass, indicating the decomposition of the protein structure. The third stage, after 400 °C, mainly involved the further degradation of residual organic matter. Additionally, at 550 °C, the residual mass of the CPI was approximately 20%, whereas the CPI-CP conjugates retained around 40%. These results align with prior research, which showed that the covalent bonding of egg white protein with gallic acid and xanthan gum enhanced its thermal stability [37].

### 3.4. Contact Angle Analysis

**Figure 5:**
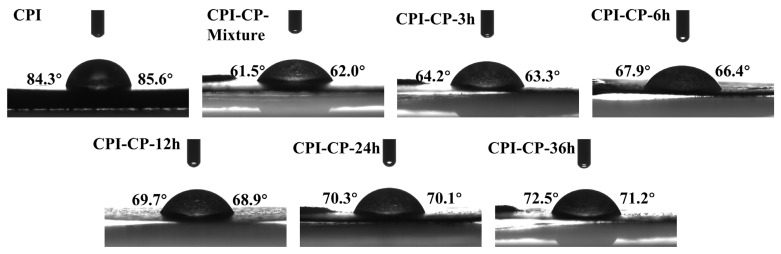
Contact angles of CPI and CPI–CP conjugates.

Contact angle measurement is a physical method commonly used to evaluate the wettability and surface properties of solid surfaces in contact with liquids [38]. For protein samples, contact angle determination is primarily employed to assess their surface hydrophilicity or hydrophobicity. The contact angles of both the CPI and CPI-CP conjugates were less than 90° (Figure 5), indicating that they exhibited surface hydrophilicity. The CPI had the largest contact angles, measuring 84.3° and 85.6°. With the introduction of the CP, the contact angle significantly decreased, with the CPI–CP–Mixture sample showing angles of 61.5° and 62.0°. This reduction can be attributed to the carboxyl and hydroxyl groups in the citrus pectin, which enhanced the hydrogen bonding with water molecules and improved the sample hydrophilicity [24]. With the progression of the Maillard reaction, the contact angle of the CPI–CP conjugates gradually increased. Notably, the CPI-CP-36 h sample displayed contact angles of 72.5° and 71.2°, significantly higher than the CPI-CP-Mixture sample. This increase may have resulted from structural changes in the CPI, which exposed more hydrophobic regions within the protein, thereby increasing the hydrophobicity of the samples [13]. A previous study demonstrated that the wet-heat Maillard reaction can improve the hydrophobicity of edible pea protein films, aligning with the findings of our study [39].

### 3.5. EAI and ESI Analysis

**Figure 6:**
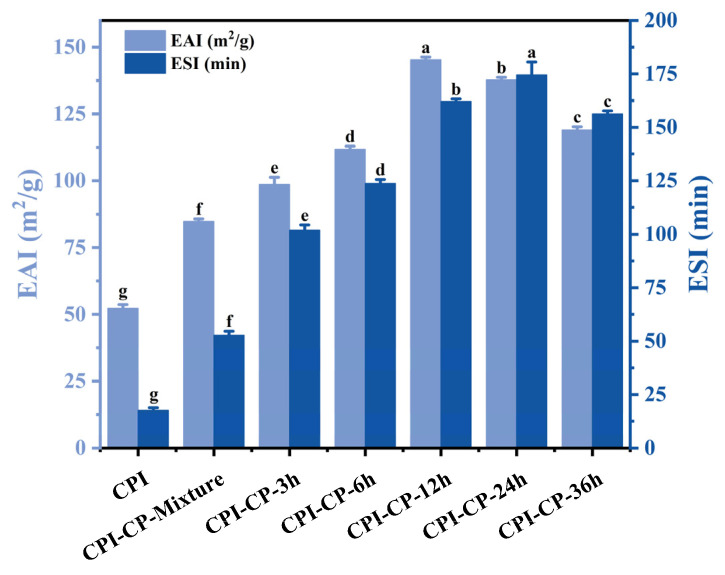
EAI and ESI of CPI and CPI-CP conjugates. Different letters indicate significant differences (*p* < 0.05).

The EAI and ESI are crucial indicators of protein emulsification performance. Both the EAI and ESI of the glycosylated CPI markedly increased with the progression of the glycosylation reaction (Figure 6). At 12 h, the EAI reached a peak of 145.33 m^2^/g, a 2.78 times improvement over the natural CPI (52.30 m^2^/g). At 24 h, the ESI attained its maximum value of 174.51 min. Similar enhancements in the EAI and ESI have been observed for pea protein isolate-xylo-oligosaccharide conjugates prepared under hydrothermal conditions. This enhancement in emulsifying properties is likely due to three main factors: (1) Glycosylation modification increased the content of hydrophilic groups in the protein molecules, making the CPI structure more flexible, improving its solubility, and facilitating its adsorption at the oil–water interface [40]. (2) Citrus pectin contributed hydroxyl groups that helped stabilize the oil and water phases, while steric hindrance reduced droplet aggregation [41]. (3) The protein-polysaccharide conjugates formed an interfacial layer, enhancing droplet dispersion and stability [42].

### 3.6. Effects of Protein Concentrations on Emulsions

**Figure 7:**
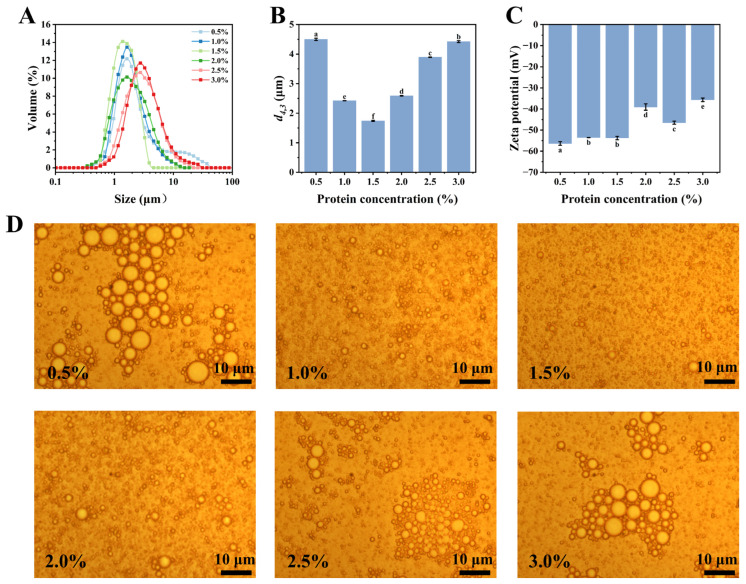
Effects of protein concentrations on emulsion properties. (**A**) Droplet size distribution. (**B**) Droplet size. (**C**) Zeta potential. (**D**) Optical micrograph. Different letters indicate significant differences (*p* < 0.05).

The protein concentration is critical in emulsion formation and stability, as insufficient protein levels fail to adequately coat oil droplets, leading to phase separation. An excessive protein concentration may reduce economic efficiency and potentially destabilize the emulsion. Figure 7 shows the influence of various concentrations of the CPI-CP conjugates on the emulsion properties. Typically, increased emulsifier concentrations enhance the oil–water interface coverage, producing smaller droplet sizes in emulsions and improving stability. Moreover, higher protein concentrations enhance the apparent viscosity of the continuous phase, limiting the Brownian motion of oil droplets and further improving emulsion stability [28]. Conversely, as the protein concentration decreases, fewer protein molecules adsorb at the oil–water interface, leading to an expansion in droplet size within the emulsion system. However, at very high protein levels, the viscosity of the continuous phase may impair emulsion dispersion during homogenization, yielding larger droplet sizes and reduced stability [43]. At a 0.5 wt% protein concentration, the emulsions formed with the CPI-CP conjugates displayed large, unstable droplets. As the protein concentration rose from 0.5 wt% to 1.5 wt%, the droplet size of the emulsion system gradually decreased, and the corresponding droplet size distribution narrowed. This can be attributed to the ability of the CPI–CP conjugates, at a certain concentration, to fully cover the oil–water interface, thereby dispersing the oil droplets effectively. When the protein concentration increased further from 1.5 wt% to 3.0 wt%, the droplet size distribution peak shifted progressively toward larger sizes, accompanied by a notable increase in d_4,3_ (*p* < 0.05), resulting in reduced emulsion stability. This instability may have arisen from steric hindrance caused by the protein-polysaccharide conjugates at higher concentrations, which disrupted the droplet size uniformity.

As shown in Figure 7C, all the emulsions displayed zeta potentials below −30 mV, indicating a substantial negative charge essential for stable emulsion formation. For protein concentrations between 0.5 and 1.5 wt%, the zeta potential remained below −50 mV. With a further increase in the protein concentration, the zeta potential rose gradually to −35.67 mV, likely due to cationic groups in the citrus pectin, which reduce the negative surface charge of oil droplets [24].

Microscopic images of emulsions at varying protein concentrations showed a pattern similar to the droplet size distribution (Figure 7D), revealing that the emulsion with a protein concentration of 1.5 wt% exhibited the smallest and most uniform droplet size. Both lower and higher protein concentrations resulted in poor droplet dispersion, leading to droplet flocculation and coalescence. Overall, the CPI-CP conjugate emulsions at 1.5 wt% protein concentration demonstrated optimal stability and performance.

### 3.7. Effects of Oil Phase Volume on Emulsions

**Figure 8:**
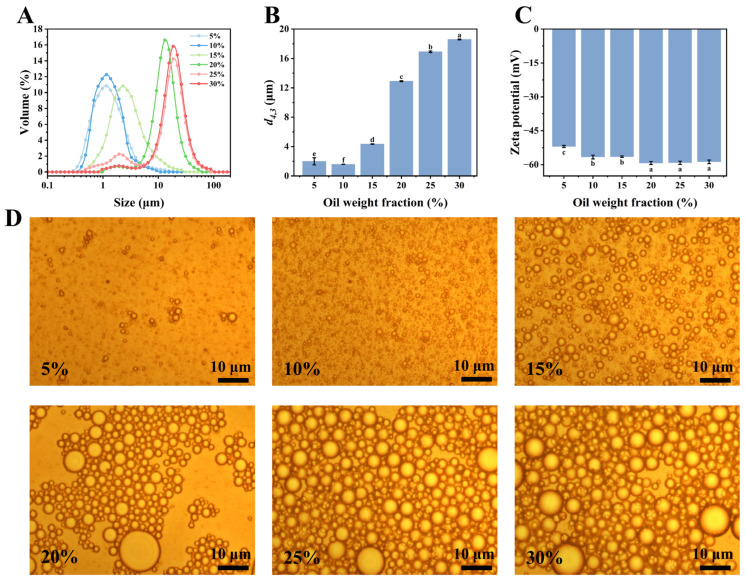
Effects of volume of oil phase on emulsion properties. (**A**) Droplet size distribution. (**B**) Droplet size. (**C**) Zeta potential. (**D**) Optical micrograph. Different letters indicate significant differences (*p* < 0.05).

As shown in Figure 8, a volume of the oil phase of 10% produced the smallest droplet size (1.59 μm). As the volume of the oil phase increased, the emulsion droplet size consistently grew, and the zeta potential gradually decreased (Figure 8C). Additionally, increasing the volume of the oil phase also enhances the viscosity and viscoelasticity of the emulsion, which contributed to its stability. Nevertheless, when the volume of the oil phase reached a critical point, the emulsifier could no longer fully cover the newly generated oil droplet interfaces, leading to droplet coalescence and, in severe cases, phase separation [44]. Figure 8D shows the optical microscopic images of the emulsions with different volume fractions of the oil phase. The microscopic images revealed a trend consistent with the droplet size distribution of the emulsions (Figure 8B). When the volume of the oil phase reached or exceeded 25%, the emulsion droplets appeared to be compressed against each other, and the droplet size increased significantly, with large droplets dominating the system. In conclusion, the emulsion stabilized by CPI-CP conjugates with a protein concentration of 1.5 wt% and a volume of the oil phase of 10% exhibited optimal stability. Consequently, these conditions were chosen for further experiments.

### 3.8. Droplet Size of Emulsions

**Figure 9:**
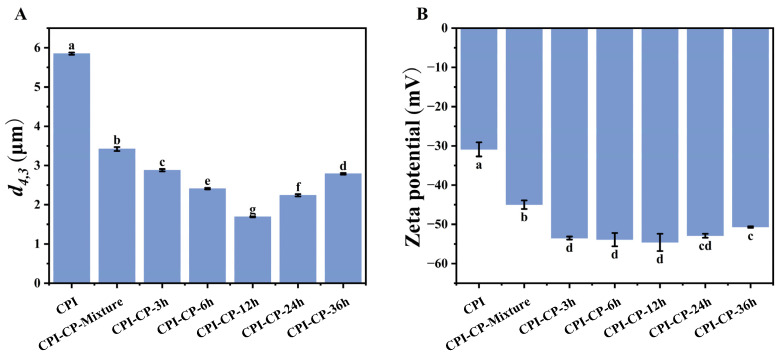
Droplet size (**A**) and zeta potential (**B**) of the emulsions prepared with CPI and CPI–CP conjugates. Different letters indicate significant differences (*p* < 0.05).

The droplet size distributions of the emulsions prepared with CPI and CPI-CP conjugates are shown in Figure 9A. The emulsions prepared with native CPI exhibited the largest droplet size, with a d_4,3_ value of 5.85 μm. This suggests that the emulsification efficiency of CPI alone may be limited, possibly due to its limited ability to effectively stabilize the oil/water interface. Conversely, the emulsions containing conjugates exhibited a notable reduction in droplet size, especially the CPI-CP-12 h sample, where the size dropped to 1.69 μm. The covalent bonding of the CP likely improved the amphiphilicity of the CPI, thereby forming smaller and more stable emulsion droplets. However, when the reaction time exceeded 12 h, the droplet size increased to 2.24 μm and 2.79 μm, respectively. This trend indicates that prolonged reaction times may lead to excessive glycosylation or structural changes in CPI-CP conjugates, such as over-crosslinking or molecular aggregation, which may impair their emulsification capabilities [45]. Additionally, the solubility of the conjugates notably declined after 12 h, which could have contributed to larger droplet sizes. This trend aligns with findings by Guo et al., who observed a decline in emulsifying properties in whey protein isolate when the Maillard reaction time was extended [35]. In conclusion, the results demonstrate that the CPI-CP-12 h sample exhibited the best emulsification capacity, producing emulsions with the smallest droplet size. Shorter reaction times may be insufficient for effective glycosylation, while excessive reaction times could lead to aggregation and diminished functionality.

### 3.9. Zeta Potential of Emulsions

The zeta potential of the emulsion prepared with CPI was measured at −30.9 mV (Figure 9B). After the Maillard reaction, the zeta potential of the emulsions prepared with the conjugates markedly decreased, with the CPI-CP-12 h sample showing a notably low value of −54.6 mV. The increased negative charge of the CPI-CP conjugates was likely due to the abundant negative charges on citrus pectin, which enhances the negative potential at the droplet surface. Furthermore, the Maillard reaction may have modified the spatial structure of the CPI, exposing additional negative charges [30]. This structural rearrangement could further influence the charge distribution on the emulsion droplet surface, resulting in an enhancement of the negative zeta potential. Emulsions stabilized mainly by electrostatic repulsion are typically stable when their zeta potential exceeds ± 30 mV [46].

### 3.10. Content of Protein Adsorption at the Interface and Thickness of the Interfacial Layer

**Figure 10:**
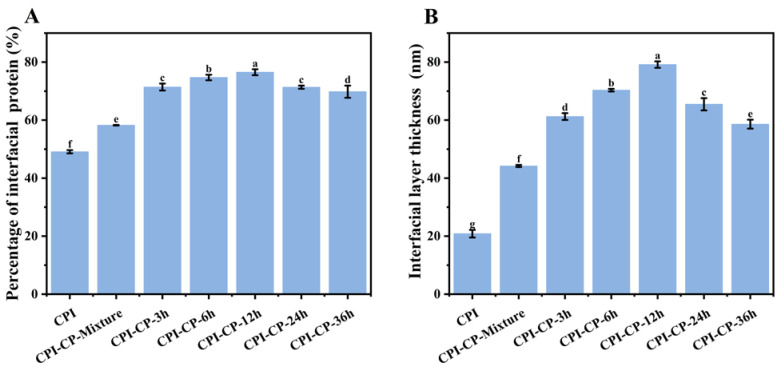
Percentage of interfacial protein (**A**) and interfacial layer thickness (**B**) of the emulsions prepared with CPI and CPI-CP conjugates. Different letters indicate significant differences (*p* < 0.05).

Protein adsorption at the oil-water interface can significantly reduce interfacial tension, thereby lowering the total free energy of the system and stabilizing the emulsion [47]. As shown in Figure 10A, introducing citrus pectin significantly increased the content of interfacial protein. Glycosylation modifications further enhanced the amount of protein adsorbed, from 58.25% to 76.51%. Concurrently, the interfacial layer thickness increased from 44.21 nm to 79.15 nm (*p* < 0.05) (Figure 10B). This increase was likely due to glycosylation modifications altering the protein conformation and enhancing the protein surface hydrophobicity. Enhanced hydrophobicity aids in more effective protein adsorption at the interface, thereby increasing the percentage of protein adsorption and the thickness of the interfacial layer [27]. Additionally, the negative charge of the pectin chains caused electrostatic repulsion with other proteins at the interface. In contrast, the volume of the sugar chains increased the physical distance between molecules, resulting in a thicker interfacial layer. The interfacial layer, which possesses higher mechanical strength and viscoelasticity, can partially mitigate droplet aggregation and coalescence, thereby enhancing emulsion stability [48].

### 3.11. Viscosity of Emulsions

**Figure 11:**
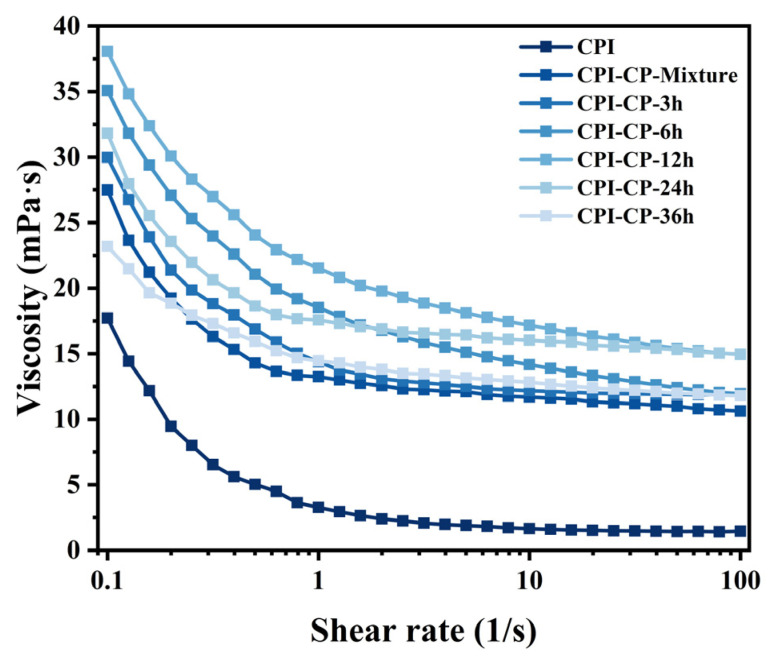
The viscosity of the emulsions is a function of the shear rate.

The viscosity of emulsions is essential for maintaining their stability. The viscosity of the emulsions prepared with CPI and CPI-CP conjugates was studied in terms of changes in the shear rate (Figure 11). Within the shear rate range of 1–100 s^−1^, the viscosity of all the emulsions progressively reduced with increasing shear rate, demonstrating the shear-thinning behavior characteristic of non-Newtonian fluids [43]. Glycosylation modifications increased the apparent viscosity of the CPI emulsions. At a shear rate of 0.1 s^–1^, the viscosity of the emulsion stabilized with CPI-CP-12 h was approximately 38.07 mPa·S. This increase was due to glycosylation enhancing the interaction forces between the protein molecules and between the emulsion droplets [29]. A high apparent viscosity can prevent phenomena such as Ostwald ripening, coalescence, and creaming [49]. However, when the reaction time exceeded 12 h, the viscosity of the CPI-CP conjugate emulsions gradually decreased. This decrease was likely due to the reduced solubility of CPI-CP conjugates, leading to a decline in the forces between the emulsion droplets.

### 3.12. Long-Term Stability of Emulsions

**Figure 12:**
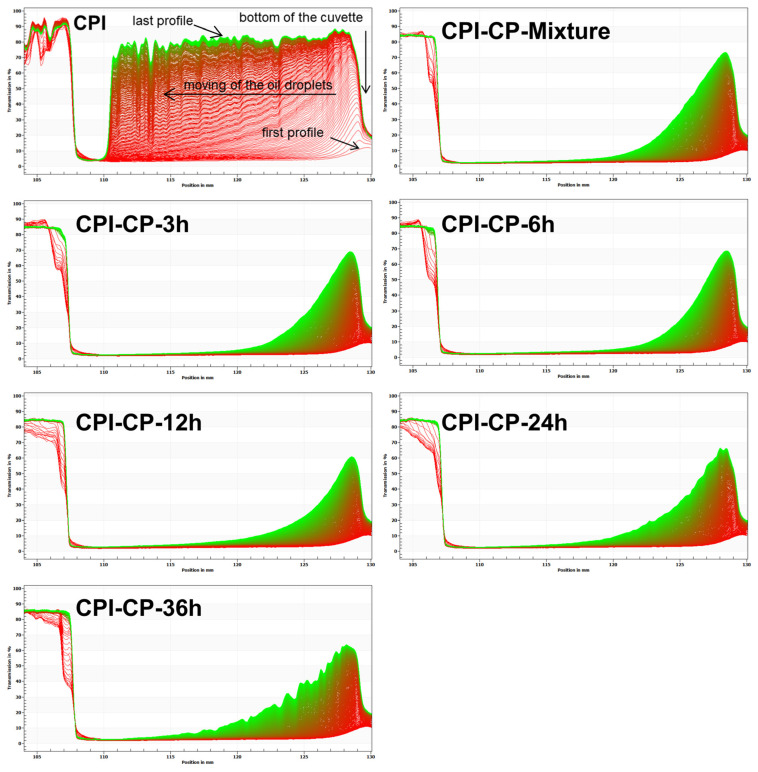
Transmission profiles of the emulsions stabilized by CPI and CPI-CP conjugates.

Due to the centrifugal forces applied in this analysis, phase separation occurred, with the lower-density oil phase migrating to the top of the sample tube and the denser aqueous phase settling at the bottom, leading to gradual stratification [50]. The transmittance changes were recorded from the initial (red) to the final transmission curve (green), enabling the visualization of particle movement within the emulsions over time. A smaller area of stacked transmission lines indicates greater stability, while a larger area signifies lower stability. Initially, all the emulsions showed a transmittance close to zero. As centrifugal force acted upon the emulsions, stratification was observed. The CPI emulsion exhibited larger gaps between the transmission curves, with the largest stacked area after centrifugation, indicating poor long-term stability (Figure 12). In contrast, the emulsion stabilized by CPI-CP-12 h showed a smaller stacked area, suggesting improved stability. This improvement was likely due to glycosylation, which augmented both the viscosity of the emulsion and protein adsorption at the interface, effectively reducing phase separation under external forces [27].

**Figure 13:**
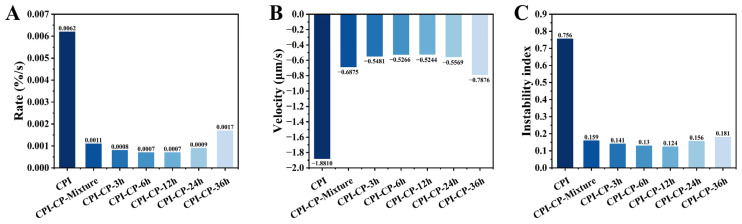
Rates of integral transmission change (**A**), migration velocity of the oil-water interface (**B**), and final instability index (**C**) for emulsions stabilized by CPI and CPI-CP conjugates.

To better compare the subtle differences in the emulsions’ stability during centrifugation, the transmission curves were integrated, and the rate of integral transmission change was calculated (Figure 13A). After glycosylation, the rate of integral transmission change decreased (0.0062–0.0007%/s). The interfacial movement rates of the emulsions were also determined to be 1.881, 0.6875, 0.5481, 0.5266, 0.5244, 0.5569, and 0.7876 μm/s, respectively (Figure 13B). A positive rate indicates droplet flocculation within the emulsion, while a negative rate reflects the ascent of oil droplets [49]. Additionally, the instability index of the emulsions was measured (Figure 13C). This dimensionless constant ranges from 0 (extremely stable) to 1 (highly unstable). The instability index increased over time, with final values of 0.756, 0.159, 0.141, 0.130, 0.124, 0.156, and 0.181, respectively. These results indicate that the CPI-CP conjugates enhanced the long-term stability of the emulsions compared to CPI alone, with the CPI-CP-12 h emulsion exhibiting the best stability. The smaller droplet sizes and viscoelastic interfacial film formed by the CPI-CP conjugates are crucial factors contributing to this enhanced long-term stability [49].

## 4. Conclusions

This study examined the impact of varying reaction durations on the physicochemical and emulsifying properties of CPI. The findings demonstrate that covalent binding between the CPI and CP resulted in conjugates characterized by larger particle sizes and increased negative charges. The Maillard reaction notably enhanced the solubility and thermal stability of the CPI. Additionally, the incorporation of citrus pectin enhanced the hydrophilicity of the CPI surface, while increased exposure of hydrophobic groups during the Maillard reaction augmented the surface hydrophobicity of the CPI. The Maillard reaction also markedly improved the EAI and ESI of the CPI. Compared with CPI alone, the emulsions prepared with CPI-CP conjugates exhibited elevated interfacial protein adsorption, increased interfacial thickness, and greater apparent viscosity, resulting in improved long-term stability, particularly for the CPI-CP conjugate produced after 12 h. However, when the reaction duration exceeded 12 h, the emulsifying properties and stability of the CPI-CP conjugates began to decline. This study offers an effective approach to enhance the functional properties of CPI, broadening the potential applications of CPI in the food industry. Future research could focus on the application of CPI-CP conjugates in various food matrices, such as dairy beverages, ice cream, and foams, to evaluate their performance under different processing conditions.

## Data Availability

The original contributions presented in the study are included in the article, further inquiries can be directed to the corresponding author.
